# Follow-up study of neuropsychological scores of infant patients with cobalamin C defects and influencing factors of cerebral magnetic resonance imaging characteristics

**DOI:** 10.3389/fnins.2022.1093850

**Published:** 2022-12-14

**Authors:** Tao Chen, Chaofan Sui, Suna Lin, Bin Guo, Yuanyuan Wang, Linfeng Yang

**Affiliations:** ^1^Department of Clinical Laboratory, Jinan Maternity and Child Care Hospital Affiliated to Shandong First Medical University, Jinan, Shandong, China; ^2^Department of Radiology, Shandong Provincial Hospital Affiliated to Shandong First Medical University, Jinan, Shandong, China; ^3^Department of Scientific Research and Foreign Affairs, Jinan Maternity and Child Care Hospital Affiliated to Shandong First Medical University, Jinan, Shandong, China; ^4^Department of Radiology, Jinan Maternity and Child Care Hospital Affiliated to Shandong First Medical University, Jinan, Shandong, China; ^5^Department of Radiology, Binzhou Medical University, Yantai, Shandong, China

**Keywords:** cblC defect, inborn error of metabolism, magnetic resonance imaging, neuropsychological test, follow-up study

## Abstract

**Purpose:**

The purpose of this study was to investigate whether baseline cerebral magnetic resonance imaging (MRI) characteristics could predict therapeutic responsiveness in patients with cobalamin C (cblC) defects.

**Materials and methods:**

The cerebral MRI results of 40 patients with cblC defects were evaluated by a neuroradiologist. Neuropsychological scores and imaging data were collected. Neuropsychological tests were performed before and after standardized treatment.

**Results:**

Thirty-eight patients initially underwent neuropsychological testing [developmental quotient (DQ)]. CblC defects with cerebellar atrophy, corpus callosum thinning and ventricular dilation had significantly lower DQs than those without (*P* < 0.05). Through a multivariate linear stepwise regression equation after univariate analysis, ventricular dilation was the most valuable predictor of lower DQs. Thirty-six patients (94.7%) underwent follow-up neuropsychological testing. The pre- and post-treatment DQ values were not significantly different (*Z* = −1.611, *P* = 0.107). The post-treatment DQ classification (normal, moderately low, or extremely low) showed nearly no change compared to the pretreatment DQ classification (*k* = 0.790, *P* < 0.001).

**Conclusion:**

Ventricular dilation, cerebral atrophy and corpus callosum thinning are the main MRI abnormalities of cblC defects, and these manifestations are significantly correlated with delayed development in children. MRI findings can be considered an important tool for determining the severity of cblC defects.

## Introduction

Cobalamin C (cblC) defects, also called combined methylmalonic aciduria and homocystinuria cblC type, are the most common inherited disorders of cobalamin metabolism, with an incidence of approximately 1/100,000 live births (Weisfeld-Adams et al., 2010). It causes impaired conversion of dietary vitamin B12 into two metabolically active forms, methylcobalamin and adenosylcobalamin. The impaired activity of these two enzymes results in the accumulation of homocysteine and methylmalonic acid as well as in the reduced synthesis of methionine (Martinelli et al., 2011). Enhanced oxidative stress due to a disturbance of glutathione metabolism has been shown in patients with cblC defects and may contribute to the underlying pathophysiology (Pastore et al., 2014). In mainland China, cblC defects are the main type of combined methylmalonic aciduria (MMA) and homocystinuria (Cui et al., 2018). Studies have found that the prevalence of cb1C deficiency in Shandong province of China was 1/3,920 from 2011 to 2014 (Han et al., 2016), however, the nationwide prevalence of cblC deficiency is unclear (Chen et al., 2022). In the case of combined defects (cblC, cblD, and cblF), both MMA and homocysteine accumulate, and these defects are vital characteristics of patients with MMA. Patients with cblC, cblD, and cblF defects are classified as having MMA combined with homocysteinemia (Fowler et al., 2008; Wang et al., 2010) accounting for 60–80% of cases in China (Wu et al., 2019), and cblC defects are the most common subtype (Carrillo-Carrasco et al., 2012).

Cobalamin C defects often cause damage to multiple body systems, especially to the central nervous system (Li et al., 2015). Although the incidence of the disease is low, the mortality and disability rates are very high (Thompson et al., 1989). This disease has diverse and non-specific clinical manifestations, such as feeding difficulties, intellectual disabilities, ataxia, abnormal muscle tone, convulsions, epilepsy, and lethargy (Fraser and Venditti, 2016; Han et al., 2016). The presence of cognitive and neurological deficits has been observed in patients with cblC defects, and these deficits include developmental delays and hyperkinetic movement disorders secondary to striatum injury (Guo et al., 2020). Patients with cblC defects usually have symptoms of neurological damage; hence, brain imaging is necessary to rule out congenital or acquired brain abnormalities, and magnetic resonance imaging (MRI) is the most valuable method for obtaining brain images because of the lack of radiation damage and its suitability for use in pediatric brain examinations (Yesildag et al., 2005). Because of the development of genetic diagnostic technologies, the diagnostic efficiency of cblC defects has greatly improved. Standardized treatment regimens for different clinical types have been established (Fraser and Venditti, 2016), and their prognosis has become a matter of widespread interest. Some previous studies have attempted to retrospectively analyze neurocognitive prognosis in patients with MMA, relying on surveys of clinical data collected across medical institutions and/or using various testing protocols to measure cognition (Baumgarter and Viardot, 1995; Hörster et al., 2009; Carrillo-Carrasco et al., 2012; Han et al., 2015).

At present, there have been many reports involving cerebral MRI characteristics of combined MMA and homocystinuria patients (Radmanesh et al., 2008; Yang et al., 2020), including detection using many functional MRI sequences (Cheng et al., 2019). However, the definite correlation between conventional MRI features and neurocognitive impairments in infant patients with cblC defects remains unclear. Follow-up studies of neurocognitive function after standardized treatment are lacking. It is unknown which MRI characteristics could predict a better recovery of neurocognitive function in patients with cblC defects. The purpose of this study was to investigate relationships between cerebral MRI characteristics and neuropsychological scores, compare changes in neuropsychological scores before and after treatment, and identify which cerebral MRI characteristics could predict a better prognosis in patients with cblC defects.

## Materials and methods

### Patient diagnosis and clinical data acquisition

Forty patients diagnosed with cblC defects were enrolled in the present study from March 2017 to December 2019. Informed consent was obtained from their guardians. This study was approved by the Ethical Committee of the Institutional Review Board (IRB) of Jinan Maternal and Child Care Hospital (201811). All experiments were performed in accordance with relevant guidelines and regulations. The diagnosis of cblC defects was made when increased plasma methylmalonyl carnitine levels or increased urine methylmalonic acid levels were detected using gas chromatography–mass spectrometry (GC/MS) and the propionate incorporation test, respectively (Radmanesh et al., 2008). The levels of propionyl carnitine (C3) and C3/acetylcarnitine (C2) were measured by tandem mass spectrometry methionine in dried blood spots. The levels of organic acids in urine were measured using GC/MS in patients with suspected cblC defects. In addition, the concentrations of total plasma homocysteine (tHcy) and blood ammonia were measured (Weisfeld-Adams et al., 2010). Genomic DNA was extracted from the peripheral blood leukocytes of patients using the phenol–chloroform method. The entire coding sequence of MMACHC was amplified by polymerase chain reaction (PCR), and the PCR products were sequenced. Variant analysis was performed using the normal MMACHC complementary DNA (NM_015506.2) sequence (Lerner-Ellis et al., 2006). Detailed genetic data and experimental data are provided in Supplementary material 1.

Magnetic resonance imaging examination and neuropsychological tests were performed before treatment. The laboratory test results, gene type, age at the time of MRI and symptoms of each patient were collected. CblC defect patients were treated with standardized treatment regimens on the basis of their different clinical types. If patients had motor system damage, sensory and motor function rehabilitation training and language cognitive ability training were carried out to facilitate the growth and development of patients. Details of the standardized treatment regimens are provided in Supplementary material 2.

### Neuropsychological assessment

The neuropsychological test used a pediatric neuropsychological development examination scale for 0- to 6-year-old patients [China neuropsychological and Behavior Scale-Revision 2016 (CNBS-R2016) scale, Capital Medical University]. This scale mainly tests the physical abilities and intelligence of infants and children in five aspects: Gross movements, fine movements, adaptability, language, and social behavior. Gross movements mainly involve the movements of the head, neck, torso, and limbs with large amplitudes; fine movements mainly refer to the movements of the hand and the hand-eye coordination ability that follows; adaptability is mainly the ability of infants and young children to analyze and synthesize external stimuli; language ability involves unique psychological activities of human beings; and social behavior is the child’s personal reaction to the real social culture.

This scale calculates the intellectual age and developmental quotient (DQ) based on age in months. Intellectual age = sum of scores in five areas/5; DQ = (intellectual age/actual age in months) × 100. The neuropsychological tests resulted in the assignment of DQs, which were classified into normal, lower and low subgroups. The classification criteria were as follows: ≥130, excellent; 115–129, intelligent; 85–114, normal; 70–84, moderately low; and ≤69, extremely low (Guo et al., 2020). Scores ≥84 were considered normal, and scores ≤84 were considered low. Follow-up tests were conducted after 6 to 12 months of treatment, and the neuropsychological scores were recorded and compared.

### Magnetic resonance imaging parameters and image interpretation

Imaging data were obtained using a 1.5-T MR scanner (Achieva, Philips Healthcare, Best, Netherlands) and a 16-channel phased-array head coil. The following sequences were performed: axial and sagittal T2-weighted imaging (T2WI) (repetition time, 2100 ms; echo time, 90–100 ms), axial T1-weighted imaging (T1WI) (TR, 568 ms; TE, 15 ms), axial T2-weighted fluid-attenuated inversion recovery (T2-FLAIR) (TR, 700 ms; TE, 115 ms), and axial diffusion-weighted imaging (DWI) (TR, 3,090 ms; TE, 99 ms; *b* = 0 to 1,000). The scanning parameters were field of view (FOV) = 230 mm × 190 mm, slice thickness = 5 mm, and slice gap = 0.5 mm. The scan time was approximately 10 min.

Images were evaluated by an experienced pediatric radiologist. The assessment of the severity of cortical atrophy and ventricular dilation was performed using the semiquantitative scale devised by Yue et al. (1997). Age-related white matter changes were assessed using the rating scale established by Wahlund et al. (2001). Then, the imaging features were defined in a binary fashion (i.e., presence or absence). Thinning of the corpus callosum was subjectively evaluated as present or absent by an experienced pediatric radiologist. To verify the accuracy of the evaluation, we measured the corpus callosum and lateral ventricle and compared the differences among healthy controls and patients with and without corresponding features. The results proved that our subjective evaluation was accurate. The specific results are presented in Supplementary material 2.

### Statistical analysis

Statistical analysis was performed using the Statistical Package for the Social Sciences (Version 21.0 for Windows; SPSS, Chicago, IL, USA). *P*-values of < 0.05 were considered to be statistically significant. First, a descriptive analysis of the 40 patients was performed. The measurement data are expressed as the median (interquartile range), and the count data are expressed as n (%). Considering that the sample size was relatively small, the Mann–Whitney *U*-test was used to compare the basic neuropsychological scores between the two groups divided by different imaging features in the baseline MRI. Then, the features that significantly influenced the DQ were selected to perform the multiple linear stepwise regression analysis. The Wilcoxon paired rank-sum test was used to compare the neuropsychological scores (DQ and five subscales), tHcy levels, blood ammonia (AMM) levels, and blood lactic acid (LAC) levels of the patients with cblC defects before and after treatment, and Bonferroni’s correction was used to avoid type I errors. Weighted *k*-values were used to assess whether the post-treatment DQ classifications were altered compared with the pretreatment DQ classifications. *K*-values of ≤ 0.4, 0.41–0.6, 0.61–0.8, and ≥0.81 indicated poor, moderate, good, and excellent agreement, respectively. To determine which image feature could potentially predict prognosis, the change value of the pre- and post-treatment DQ scores was considered a marker for evaluating prognosis, and the Mann–Whitney *U*-test was used to compare the difference in the change values between the two groups into which patients were divided based on baseline MRI image features.

## Results

Of the 40 patients (29 males and 11 females), 19 patients were diagnosed by genetic screening shortly after birth, and 21 patients were diagnosed by blood and genetic tests after exhibiting suspicious symptoms. In these 40 patients, the age at which they began to exhibit symptoms was 7.05 (3.00, 20.50) months. The presenting symptom age of patients who were diagnosed by genetic screening was 18.00 (3.00, 30.0) months, while the presenting symptom age of patients who were diagnosed by blood and genetic tests after exhibiting suspicious symptoms was 5.00 (2.05, 11.00) months. The difference in onset age was not statistically significant between these two groups (*Z* = 1.939, *p* = 0.052). In these 40 patients, the age at which the baseline MRI scan was conducted was 9.00 (3.25, 23.75) months, usually several weeks after symptom onset. The first neuropsychological test and MRI examination usually occurred on the same day or within 2 days. When the pediatrician received the results within 2 days, the treatment started immediately; therefore, neuropsychological test time, MRI examination time and treatment initiation time could be considered approximately one time point. Neuropsychological evaluations were performed at intervals of 6 to 12 months before and after treatment, and the age at follow-up DQ evaluation was 18.50 (13.12, 30.75) months. These patients’ symptoms included seizures, developmental regression, hypotonia, psychomotor delay, recurrent vomiting, respiratory distress, agitation, coma, lethargy, growth retardation, and poor feeding. Thirty-eight cblC defect patients received neuropsychological testing, and 2 patients (5.3%) were not successfully tested due to non-compliance. Thirty-six patients (94.7%) were followed up and received a second neuropsychological test after 6 to 12 months of standardized treatment.

All patients included in this study underwent routine MRI examinations. The main MRI feature details are shown in Table 1. The patients with cblC defects were divided into two groups based on the presence or absence of a particular MRI feature, and differences in DQ scores were compared. Because the number of samples in some groups was small and the data were not normally distributed, the rank-sum test of two independent samples was used. The comparison results are shown in Table 1. The cblC defect patients with characteristics of cerebellar atrophy, corpus callosum thinning and ventricular dilation had significantly lower DQs than the patients without these characteristics (*P* < 0.05). Using Spearman bivariate correlation analysis, we found that the DQ value negatively correlated with tHcy (*r* = −0.333, *p* = 0.041). However, AMM and LAC had no relationships with the DQ values (*r* = 0.123, *p* = 0.464; *r* = 0.218, *p* = 0.189). After univariate analyses, the factors cerebellar atrophy, corpus callosum thinning, ventricular dilation and tHcy were entered into the multivariate linear stepwise regression equation. Only the parameter of ventricular dilation was included in the final equation. The results showed that the patients with ventricular dilation had a lower DQ, as shown in Table 2. In addition to the change in DQ scores before and after treatment between the two groups (divided by the presence or absence of a particular MRI feature in the baseline scan), the results showed that there were no significant differences in the DQ value changes between these two groups (*P* > 0.05).

**TABLE 1:** Cerebral MRI characteristics in patients with cblC defects and comparison of DQ among different groups.

MRI characteristics	Descriptive MRI image feature analysis of 40 patients			Basic DQ comparison between two group based different MRI image features				Comparison of the difference of two time-point DQ values based different MRI image features			
			
		*N* (40)	%	*N* (38)	Median (interquartile range)	*Z*	*P*	*N* (36)	Median (interquartile range)	*Z*	*P*
Cerebral sulcus widened	No	24	60.0	22	76.90 (46.67, 87.63)	−0.325	0.759	20	0.40 (−2.5, 6.58)	−0.653	0.519
	Yes	16	40.0	16	71.40 (50.00, 85.70)			16	0.95 (−2.35,11.85)		
Cerebellar atrophy	No	32	80.0	30	78.95 (60.90, 87.63)	−2.166	0.028	28	0.55 (−2.5, 9.38)	−0.133	0.896
	Yes	8	20.0	8	44.5 (32.15, 73.95)			8	0.90 (−2.35, 2.25)		
Brainstem thinning	No	36	90.0	34	76.95 (60.90, 86.25)	−2.759	0.051	32	0.30 (−3.0, 7.73)	−1.435	0.157
	Yes	4	10.0	4	33.70 (20.55, 38.45)			4	2.10 (1.50,31.35)		
Corpus callosum thinning	No	21	52.5	20	85.50 (70.48, 88.95)	−3.070	0.002	19	0.10 (−3.0, 3.80)	−1.363	0.175
	Yes	19	47.5	18	56.95 (35.25, 76.28)			17	1.80 (−1.90, 19.95)		
Cortical atrophy (sulcal widening)	No	25	62.5	23	79.60 (63.90, 88.00)	−1.643	0.101	22	0.4 (−2.5, 6.58)	−0.114	0.911
	Yes	15	37.5	15	69.00 (36.80, 83.30)			14	0.95 (−2.35, 11.85)		
Subcortical/periventricular white matter change	No	12	30.0	10	66.95 (42.10, 80.20)	−1.293	0.196	8	3.20 (0.25, 16.85)	−1.389	0.165
	Yes	28	70.0	28	77.55 (50.00, 87.88)			28	0.35 (−4.28, 6.58)		
Ventricular dilation	No	28	70.0	26	82.50 (69.43, 88.20)	−3.360	0.000	24	0.15 (−2.5, 5.38)	−1.695	0.090
	Yes	12	30.0	12	44.50 (24.38, 69.75)			12	2.1 (−2.15, 26.78)		
Myelination abnormality	No	17	42.5	15	75.60 (51.90, 85.90)	−0.164	0.883	14	1.15 (−0.03, 15.30)	−1.347	0.180
	Yes	23	57.5	23	74.20 (48.70, 87.50)			22	0.35 (−3.43, 4.73)		
Internal capsule change	No	36	90.0	34	74.85 (49.68, 86.83)	−0.428	0.697	32	0.70 (−2.5, 7.73)	−0.201	0.865
	Yes	4	10.0	4	75.60 (40.20, 82.65)			4	−0.35 (−2.45, 30.78)		
Signal change consistent with focal infarct	No	37	92.5	35	75.50 (50.00, 86.40)	−0.217	0.839	33	0.50 (−2.0, 7.65)	−0.344	0.746
	Yes	3	7.5	3	39 (50, 92)*			3	−4.70 (1.40, 28.5*		
Abnormal signal in basal ganglia	No	39	97.5	37	75.50 (49.35, 86.50)	−0.821	0.526	35	0.50 (−3.0, 7.50)	−1.493	0.167
	Yes	1	2.5	1	50◆			1	28.50◆		

*There were only three patients in this group, and no interquartile range is shown. All values are shown in the table. ◆There was only one patient in this group, and no interquartile range is shown. The values are shown in the table.

**TABLE 2:** Multiple linear stepwise regression equation.

Variable	Non-normalized B	Standard error	Normalized B	*t*	*p*
C	75.72	3.960		19.121	0.000
Ventricular dilation	−28.890	7.047	−0.564	−4.100	0.000

In this study, 38 patients received neuropsychological testing before treatment [DQ, 74.85 (49.68, 86.45)], and of these patients, 36 were followed up after 6–12 months of treatment [DQ, 78.00 (62.40, 85.75)]. There were no significant differences in DQs before and after treatment. Using the DQ subscales, all the subscores from the follow-up evaluation were higher than those before the treatment. To avoid type I error, Bonferroni’s correction was used, and finally, no difference between these subscores reached the level of statistical significance (see Table 3 for details). Weighted *k*-values were used to compare the changes in DQ classification pre- and post-treatment (*k*-values = 0.790, *P* < 0.001; Table 4). After treatment, the classification of 31 patients remained unchanged; the classification of 5 patients increased to a higher rank, and the classification of only one patient decreased to a lower rank.

**TABLE 3:** Comparative analysis of DQ before and after treatment in patients with cblC defects.

DQ scale and subscales	DQ-first (*n* = 38) median (interquartile range)	DQ follow-up (*n* = 36) median (interquartile range)	Statistics	
			*Z*	*P* *
DQ	74.85 (49.68, 86.45)	78.00 (62.40, 85.75)	−1.611	0.107
Gross movements	73.75 (41.80, 85.60)	79.85 (59.35, 90.55)	−2.211	0.027
Fine movements	72.50 (46.05, 86.18)	71.50 (59.95, 84.83)	−0.744	0.457
Adaptability	74.60 (42.73, 87.35)	79.40 (60.00, 89.93)	−2.453	0.014
Language	73.75 (50.73, 88.50)	74.45 (61.50, 81.18)	−0.368	0.713
Social behavior	76.90 (50.98, 87.95)	76.40 (61.00, 88.00)	−0.838	0.402

DQ-first, DQ obtained before treatment; DQ-follow up, DQ obtained after 6 months of treatment. The Wilcoxon paired rank sum test was used to compare the neuropsychological scores (DQ and five subscales) of the cblC defects before and after treatment, and the Bonferroni’s method was used for correction to avoid type-I error. *Statistical test level, *P* < 0.05/6 = 0.008 (Bonferroni’s correction).

**TABLE 4:** Kappa test of DQ classification reproducibility before and after treatment in patients with cblC defects.

DQ-first	DQ-follow up			Total
	Low	Lower	Normal	
Low	10	3	0	13
Lower	0	12	2	14
Normal	0	1	9	9
Total	10	15	11	36

DQ: 84–114, normal; 70–84, moderately low; ≤69, extremely low. Kappa = 0.790, *P* < 0.001.

In this study, the tHCy, AMM and LAC values of 40 cblC defect patients after treatment were significantly lower than those before treatment (see Table 5 for details).

**TABLE 5:** Comparative analysis of blood examination before and after treatment in patients with cblC defects.

Variables	Before treatment	After treatment	Statistics	
			*Z*	*P*
tHcy (μmol/L)	61.95 (51.05, 93.53)	30.85 (21.50, 37.10)	−5.511	0.000
AMM (μmol/L)	49.50 (36.13, 65.66)	26.55 (22.83, 30.63)	−5.511	0.000
LAC (mmol/L)	4.08 (3.04, 6.04)	2.68 (2.37, 2.99)	−4.967	0.000

## Discussion

This study demonstrated that the DQs of cblC defects with cerebellar atrophy, thinned corpus callosum, and ventricular dilation were significantly lower than those without these features; the results of multiple stepwise linear regression indicated that the occurrence of ventricular dilation had the greatest effect on the DQ scores. By comparing the changes in DQ before and after treatment, we found that the DQ of the cblC defect did not significantly decrease. After standardized treatment, the DQ of most patients increased. However, among the abnormal features detected by routine MRI, no features could predict better prognosis.

**FIGURE 1:**
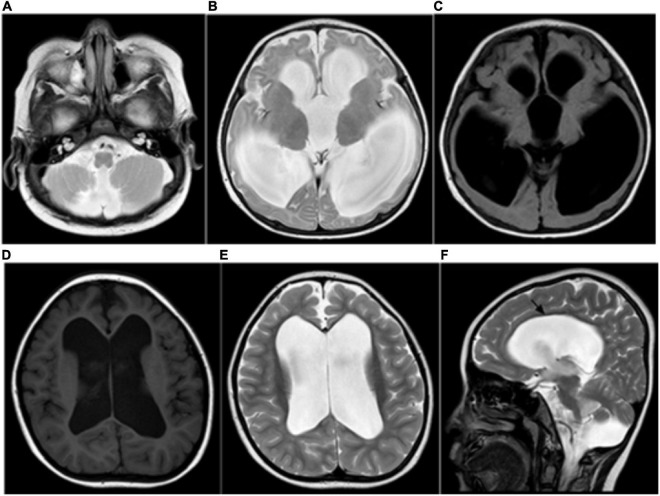
**(A–C)** Ventricular dilation and a small cerebellar vermis, consistent with the Dandy–Walker variant. **(D–F)** MRI showing severe ventricular dilation and corpus callosal thinning (black arrow, sagittal T2WI).

Magnetic resonance imaging has become the preferred imaging method for examining newborns or infants because it does not involve radiation and is characterized by high tissue contrast and high spatial resolution. In this study, forty cblC defects underwent routine MRI examinations. The most common imaging findings in the patients in this study were corpus callosum thinning (47.5%), cerebral sulcus widening (40%), cortical atrophy (37.5%), subcortical/periventricular white matter changes (35%), and ventricular dilation 30%) (Figures 1A–F). These findings are similar to previous studies (Gebarski et al., 1983; Korf et al., 1986; Heidenreich et al., 1988; Desousa et al., 1989; Brismar and Ozand, 1994; Enns et al., 1999; Biancheri et al., 2001; Pui and Ahmad, 2002; Takeuchi et al., 2003; Yesildag et al., 2005; Radmanesh et al., 2008).

Furthermore, we found that the DQ of cblC defect patients with cerebellar atrophy, thinned corpus callosum, and ventricular dilation was significantly decreased. The results of multivariate linear stepwise regression analysis showed that the occurrence of ventricular dilation had the largest impact on the brain development of patients with cblC defects. Ventricular dilation, cerebral sulcal widening and cerebellar atrophy are vital MRI signs reflecting neuropsychological developmental disorders in cblC defects. Some researchers have also considered that screening for inherited metabolic diseases should be immediately conducted when pediatric patients with cblC defects develop progressive and refractory hydrocephalus (Zhang et al., 2019). Previous studies have reported that ventricular dilation might be related to intracerebral vascular stiffness (Greitz et al., 2010). The toxic effects of high concentrations of cysteine metabolites on the vascular wall are the main cause of vascular endothelial damage (Herrmann and Knapp, 2002), which could lead to a decrease in the ploidy of arteries. The cause of cerebral atrophy is a reduction in white matter volume, which is known to stunt growth. Cerebral atrophy can directly affect the development of the cerebral nervous system in newborns or infants (Radmanesh et al., 2008). However, follow-up images of cblC defects after treatment were lacking, and we could not explain whether the severity of these signs would decrease after treatment. Longitudinal research is needed, and we will further study this question.

Currently, the most widely used intelligence test assessment method worldwide is the Wechsler Intelligence Scale (WISC); this scale can measure the general level of integrated intelligence (IQ) and different aspects of IQ, namely, language IQ and operational IQ, and this scale can be used to assess children (aged 6–16 years old) (Na and Burns, 2016). This testing method has been widely used to assess the brain development of children. Many studies have explored correlations between scale scores and related MRI indicators (Nestor et al., 2015; Xia et al., 2016). However, the children in this study were approximately 0–6 years old, and the WISC was not suitable for patients with cblC defects in our study. In our study, we used a scale suitable for use with 0- to 6-year-old patients [(CNBS-R2016) scale] to assess the neuropsychological development of pediatric patients when MRI examinations were performed. The DQ was calculated to evaluate the neuropsychological development status of the cblC defect patients. This scale not only used the DQ to evaluate the rate of development of the child’s intelligence but also used intellectual age to indicate the child’s development level, which provides a reliable early diagnostic basis for abnormal intelligence or developmental delay. This scale is currently widely used in China and is more suitable for evaluating the developmental characteristics of Chinese children. The scale was also applied to our recent assessment of brain development in cblC defects (Guo et al., 2020). Abnormal MRI findings provide a visual basis for understanding morphological changes associated with the impaired neuropsychological development of cblC defects. Our results suggested that damage to the central nervous system and delay of neuropsychological development in children with cblC defects can be reflected by MRI findings.

We used MRI images to analyze the abnormal brain structure of patients with cblC defects and attempted to identify specific features that were closely related to the prognosis of patients, which has not been done in previous related research. Our research team is part of the national diagnosis and treatment center for genetic and metabolic diseases. After the onset of cblC defects, MRI examination and neuropsychological evaluation were performed in a timely manner (see Table 1 for details). After receiving clinical and laboratory examinations, patients with cblC defects were treated in a timely manner according to their genotypes and received a periodic follow-up evaluation after 6 to 12 months of treatment. Neuropsychological scores can objectively reflect the changes in the conditions of patients with cblC defects before and after treatment, which makes it possible for us to identify specific MRI features that could predict the prognosis of patients with cblC defects.

In this study, 36 patients underwent neuropsychological testing after standardized treatment. It was found that the DQ obtained from the two tests did not show significant changes. We further conducted evaluations using DQ subscales and found that the scores for gross movement, fine movement, adaptability, language, and social behavior all increased after a 6–12 months standardized treatment. To ensure the accuracy of the multiple comparisons and to avoid type I errors, Bonferroni’s correction was adopted. Although the increases in these scores did not reach the level of significance, the results were still encouraging. The kappa value indicated that the DQ classification of 31 patients was stable; the classification of 5 patients improved to a higher rank, and the classification of only one patient decreased to a lower rank. Such follow-up results suggest that standardized treatment in patients with cblC defects can maintain stable DQ levels with a tendency to improve. If patients continue to receive standardized treatment, the DQ may significantly improve. However, long-term follow-up tests and MRI examinations are needed to understand subsequent changes in DQs in cblC defects. From another perspective, it was also suggested that regardless of what abnormal MRI manifestations were observed, patients would improve after standardized treatment; this would stabilize the patient’s condition, and the DQ could be improved to a certain extent. We are optimistic that with the extension of the treatment time, patients will show a significant improvement. Therefore, pediatricians and parents can have increased confidence in continuing treatment, which is necessary in our clinical practice.

The short follow-up period is a limitation of this study. However, clinicians and parents are eager to know whether the patients have improved, which is important for them to establish confidence in the next treatment. In future studies, we should continue to follow up with the patients included in this study and obtain neuropsychological scores and MRI data at multiple time points to better explore the prognosis of cblC defects and their influencing factors. No comparisons were made across the different genetic subgroups regarding the imaging manifestations. Furthermore, we will continue to expand the sample size to observe the different genetic subgroups of MMA and homocystinuria patients. Various functional imaging sequences, such as magnetic resonance spectroscopy, have been applied to the study of patients with combined MMA and homocystinuria (Cheng et al., 2019). We will also apply multimodal MRI in these studies to obtain additional morphological and functional data.

## Conclusion

Ventricular dilation, cerebral atrophy and corpus callosal thinning are the main MRI abnormalities of cblC defects, and these manifestations are significantly correlated with delayed development in children. However, in routine MRI findings, no abnormal features were found to have the ability to predict the prognosis of cblC defects. After standardized treatment, the DQ and subscale scores increased in most of the patients, although these differences did not reach significance. MRI findings can be considered an important tool for evaluating the neuropsychological development of cblC defects.

## Data availability statement

The original contributions presented in this study are included in the article/Supplementary material, further inquiries can be directed to the corresponding author.

## Ethics statement

The studies involving human participants were reviewed and approved by the Ethical Committee of the Institutional Review Board (IRB) of Jinan Maternal and Child Care Hospital (201811). Written informed consent to participate in this study was provided by the participants’ legal guardian/next of kin.

## Author contributions

TC and CS wrote the main manuscript text. CS prepared the figure. SL did the statistical analysis. BG and YW prepared the clinical data and imaging data. LY revised the main manuscript text. All authors reviewed the manuscript.
