# Supplementary material for: Follow-up study of neuropsychological scores of infant patients with cobalamin C defects and influencing factors of cerebral magnetic resonance imaging characteristics
Source: Front Neurosci. 2022 Dec 14;16:1093850. doi: 10.3389/fnins.2022.1093850 (PMC9795007; doi:10.3389/fnins.2022.1093850)
Supplement: Supplementary file 2 [file Data_Sheet_2.DOCX]

**Supplemental Materials 2**

**Standardized treatment regimens for cblC defects**

The patients with cblC defects were treated with hydroxocobalamin (OHCbl) (1 mg/day, intramuscular injection), betaine (100-500mg/kg/day, oral administration), folic acid (5-10mg/day, oral administration),vitamin B6 (10-30mg/day, oral administration), sodium benzoate (150-250mg/kg/day, improve hyperammonemia), levocarnitine (50mg/kg/day, oral administration), low protein and high-energy diet to reduce the accumulation of toxic metabolites.

Fifty-two healthy children were included as healthy controls (HCs). The MMA patients and HCs were matched for gender and age.

| Group | N | Age in months  Median (interquartile range) | Z | P |
| --- | --- | --- | --- | --- |
| HC | 52 | 8 (3.25, 22.5) | -0.165 | 0.869 |
| MMA patients | 43 | 7.1 (3.0, 23) |  |  |

|  | Gender |  | Total |
| --- | --- | --- | --- |
|  | Male | Female |  |
| HC | 30 | 22 | 52 |
| MMA patients | 35 | 14 | 49 |
| Total | 65 | 36 | 101 |

*χ^2^*=2.075，P=0.150

**1. Objective verification of subjective evaluation of ventricular dilation**

We measured the width of the lateral ventricles using six diameters, namely, the width of the anterior horn, body and posterior horn of the right and left lateral ventricle, and used the sum of these six diameters to estimate the degree of expansion of the lateral ventricle. See the **Figures** below for the method. We found that there was a significant difference among HCs, MMA patients with ventricular dilation, and MMA patients without ventricular dilation.


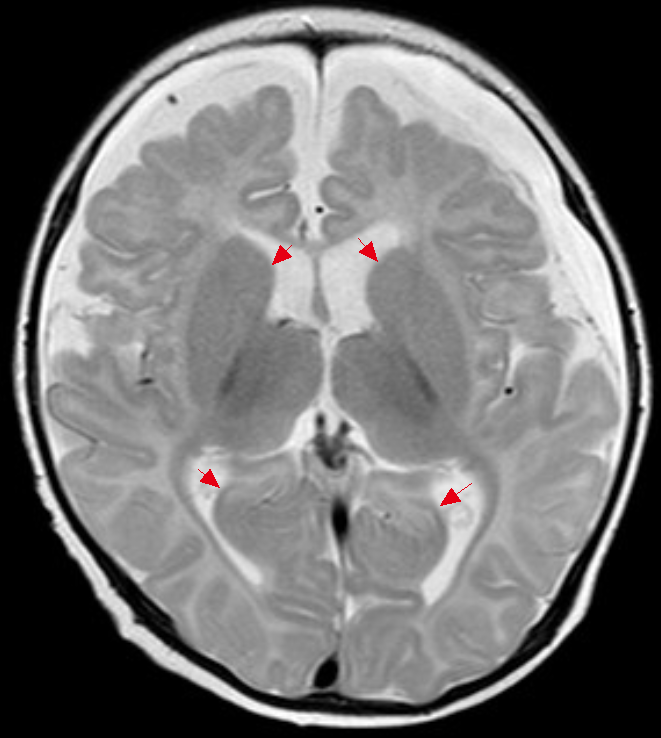

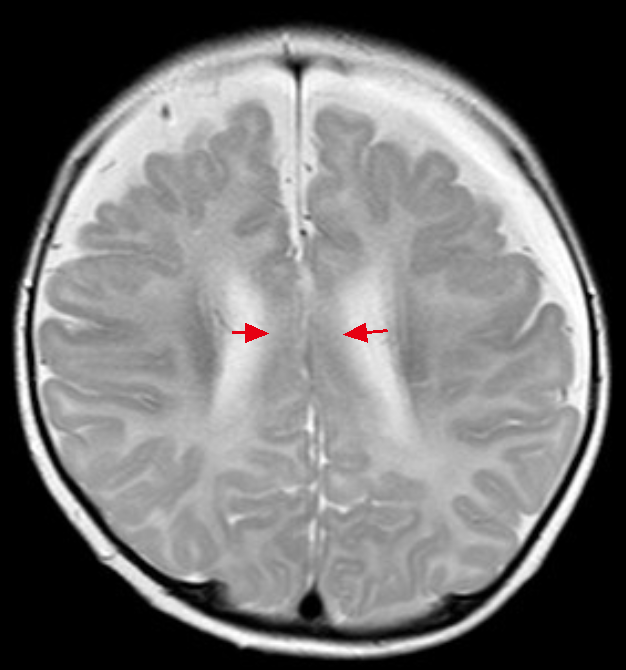


| Group | N | Median (mm)  (interquartile range) | Z | P |
| --- | --- | --- | --- | --- |
| HCs | 52 | 34.15 (30.2, 39.58) | 29.852 | ≤0.001 |
| MMA patients without ventricular dilation | 26 | 36.65 (33.0, 43.20) |  |  |
| MMA patients with ventricular dilation | 11 | 98.6 (65.8, 139.50) |  |  |
| Total | 89 | 37.0 (32.1, 43.10） |  |  |

The Kruskal-Wallis H test was used to compare the diameters among the three groups. When pairwise comparisons were conducted, the significance threshold was modified to P<0.017. HCs vs MMA patients with ventricular dilation, P≤0.001; HCs vs MMA patients without ventricular dilation, P=0.090; MMA patients without ventricular dilatation vs MMA patients with ventricular dilatation, P≤0.001.

**2. Objective verification of the subjective evaluation of corpus callosum thinning**

We measured the thickness of the corpus callosum using three diameters, namely, the width of the genu, body and splenium of the corpus callosum; we used the sum of these three diameters to estimate the degree of thinning of the corpus callosum. See the **Figure** below for the method. We found a significant difference among HCs, MMA patients with corpus callosum thinning, and MMA patients without corpus callosum thinning.


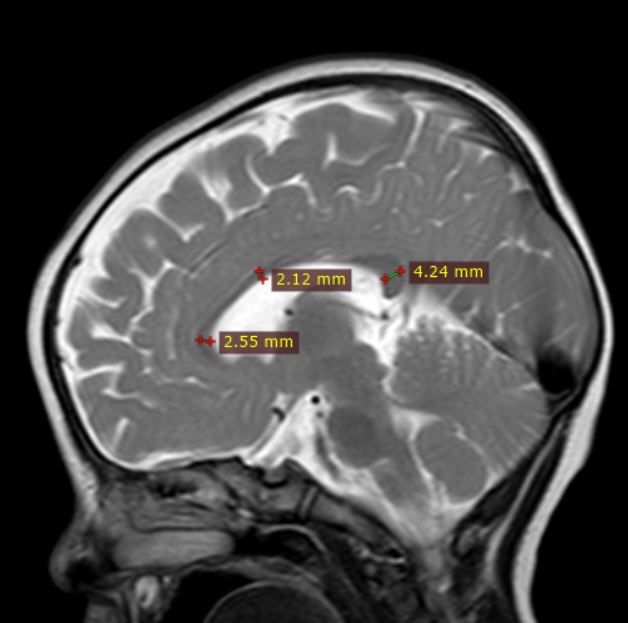


| Group | N | Mean±SD (mm) | | F | P |
| --- | --- | --- | --- | --- | --- |
| HC | 52 | | 13.70±5.19 | 6.779 | 0.002 |
| MMA patients without corpus callosum thinning | 22 | | 13.81±4.69 |  |  |
| MMA patients with corpus callosum thinning | 15 | | 8.75±3.04 |  |  |
| Total | 89 | | 12.89±5.09 |  |  |

ANOVA testing was used to compare the callosal thickness among the three groups. When pairwise comparisons were conducted, the least significant difference (LSD) test was adopted. HCs *vs* MMA patients with corpus callosum thinning, P=0.001; HCs *vs* MMA patients without corpus callosal thinning, P=0.929; MMA patients without corpus callosum thinning *vs* MMA patients with corpus callosum thinning, P=0.002.

The thickness of the corpus callosum was significantly correlated with the age of the subjects (r=0.676, P≤0.001). Therefore, the specific quantitative value could not be used to judge whether there was corpus callosum thinning because the standards would differ among children of different ages; instead, callosal thickening was judged by a senior pediatric radiologist.
